# Structure of Staphylococcal Enterotoxin E in Complex with TCR Defines the Role of TCR Loop Positioning in Superantigen Recognition

**DOI:** 10.1371/journal.pone.0131988

**Published:** 2015-07-06

**Authors:** Karin E. J. Rödström, Paulina Regenthal, Karin Lindkvist-Petersson

**Affiliations:** Department of Experimental Medical Science, Lund University, BMC C13, 22 184, Lund, Sweden; University of Washington, UNITED STATES

## Abstract

T cells are crucial players in cell-mediated immunity. The specificity of their receptor, the T cell receptor (TCR), is central for the immune system to distinguish foreign from host antigens. Superantigens are bacterial toxins capable of inducing a toxic immune response by cross-linking the TCR and the major histocompatibility complex (MHC) class II and circumventing the antigen specificity. Here, we present the structure of staphylococcal enterotoxin E (SEE) in complex with a human T cell receptor, as well as the unligated T cell receptor structure. There are clear structural changes in the TCR loops upon superantigen binding. In particular, the HV4 loop moves to circumvent steric clashes upon complex formation. In addition, a predicted ternary model of SEE in complex with both TCR and MHC class II displays intermolecular contacts between the TCR α-chain and the MHC, suggesting that the TCR α-chain is of importance for complex formation.

## Introduction

T cell activation is a fundamental event in the immune response, which requires T cell receptor (TCR) recognition of a peptide presented by the major histocompatibility complex (MHC) [1]. The T cell receptor is a transmembrane protein with an extracellular antigen-binding domain, consisting of an α- and a β-chain, each comprising one variable (TRAV and TRBV) and one constant (TRAC and TRBC) domain [2]. There are three loops, the CDR1-3, in the variable domains of TCR that predominantly recognize the peptide-MHC complex. A fourth loop, HV4, is also variable, but generally not used for antigen recognition [3]. The HV4 loop has, however, been suggested to be important when T cells are activated by certain bacterial toxins, called superantigens (SAgs) [4, 5]. Superantigens are immune stimulatory toxins that bind directly to TCR and MHC as unprocessed proteins, and hence prevent the TCR from recognizing the peptide presented by MHC [6–8]. By this cross-linking event, superantigens are capable of evoking an immune response of large proportions, resulting in host disease [9]. The superantigens produced by *Staphylococcus aureus* and *Streptococcus pyogenes* are divided into five evolutionary groups (I-V), depending on sequence similarity, and each group has structurally diverse ways of engaging TCR and MHC class II [10]. Staphylococcal enterotoxin E (SEE) belongs to group III, which generally has one binding site to TCR, to the TRBV domain [11], and two distinct binding sites to MHC class II [12–15]. The first MHC binding site is located in the N-terminal domain of the SAg, which binds to the α-chain of MHC, with relatively low affinity [15], and the other is zinc bridged and located in the C-terminal domain of the SAg, which engages the β-chain of MHC with high affinity [14]. SEE has until now evaded crystallographic studies, but the structure of the closely related superantigen SEA has been determined [16]. Still, neither SEA, nor SEE has been structurally elucidated in complex with a T cell receptor, even though structures of these complexes are of particular interest since a potential drug for cancer treatment consists of a chimera of these two superantigens [11, 17, 18].

Here, we present the structures of a T cell receptor, both by itself and in complex with staphylococcal enterotoxin E. The SEE-TCR structure describes the very first interface between a group III superantigen and its TCR. Upon superantigen engagement, the T cell receptor undergoes no global structural changes, but there are several smaller movements of the TCR-loops and a larger conformational change in the HV4 loop upon complex formation. This suggests that SEE-recognition is dependent on flexibility in the T cell receptor antigen binding surface. Moreover, structural alignment of several TRBVs suggests that the conformation of the CDR2 loop is particularly important for SEE recognition. In addition, we generated a TCR-SEE-(MHC)_2_ model, which shows that TCR, SEE and one MHC form a triangular complex with an additional interface between TRAV and the β-chain of MHC, as observed for related superantigens [19].

## Materials and Methods

### Protein production and purification

The T cell receptor with variable domains TRAV22/TRBV7-9 was prepared as described previously [20, 21], apart from minor changes stated here. Expression was carried out in *Escherichia coli* BL21 (DE3) Star (Invitrogen). Inclusion bodies were solubilized in 50 mM Tris-HCl pH 8.0, 6 M guanidinium chloride, 100 mM NaCl, 10 mM EDTA, 5 mM DTT and then refolded in 100 mM Tris-HCl pH 8.0, 5 M urea, 400 mM L-arginine, 0.83 mg/l cysteamine. Purification was done by anion exchange chromatography on an ÄKTA explorer (GE Healthcare), with a Resource Q 6 ml column (GE Healthcare), followed by size exclusion chromatography on a Superdex 200 column (GE Healthcare) in TBS buffer. Purified staphylococcal enterotoxin E, a gift from Active Biotech Research AB, was prepared according to a previously published protocol [22].

### Crystallisation and structure determination

The TCR was crystallized by vapour diffusion at 5.0 mg/ml in 22% PEG 2,000 MME, 0.1 M ammonium chloride pH 8.5 and 0.1 M NaCl. For cryo protection, 20% glycerol was used and data were collected at 100 K and 1.000 Å at ESRF beamline ID 23–1 (Table 1). A high resolution pass with an oscillation angle of 0.1°, using helical oscillation, and a low resolution pass with an oscillation angle of 0.5° on a single location was collected from a single crystal. The TCR data were indexed, integrated and merged with XDS [23] and aimless [24, 25], within autoPROC [26]. Subsequently, the TCR structure was solved using molecular replacement in Phaser [27], with TRAV, TRAC, and TRBC domains from 2IAL [28] and TRBV domain from 2DX9 [29] as search models. Differing amino acids were initially omitted and then built using Buccaneer [30, 31]. Refinement was carried out in autoBUSTER [32] and refmac5 [33] with manual modeling in Coot [34], with anisotropic B-factors for protein atoms and isotropic B-factors for solvent molecules. Finally, a composite omit map was generated using CNS [35] with 5% of the structure omitted. The final TCR model comprised residues 2–203 in TCRα and 3–243 in TCRβ, along with one glycerol molecule and 309 waters. Ramachandran statistics, calculated with SFCHECK [36], for the TCR structure were 91.9% in preferred, 7.1% in allowed, and 1.0% in generously allowed regions.

**Table 1:** Data collection and refinement statistics.

	TCR	SEE-TCR
**Data collection**		
Space group	P2_1_	P2_1_2_1_2_1_
Cell dimensions		
*a*, *b*, *c* (Å)	39.07, 79.75, 69.03	63.13, 78.54, 180.8
*α*, *β*, *γ* (°)	90, 105.9, 90	90, 90, 90
Resolution (Å)	39.9–1.34 (1.43–1.34)	47.81–2.50 (2.60–2.50)
No. reflections / unique	263,650 / 87,661	140,612 / 31,731
*R*_merge_	0.061 (0.703)	0.075 (0.774)
*I / σI*	20.2 (3.5)	10.6 (2.0)
Completeness (%)	97.1 (89.5)	99.5 (98.2)
Redundancy	3.0 (2.2)	4.4 (4.4)
**Refinement**		
Resolution (Å)	1.35	2.50
*R*_work_ / *R*_free_	0.1557 / 0.1929	0.2463 / 0.2551
No. atoms		
Protein	3695	4862
Zinc	-	1
Sodium	-	2
Glycerol	6	-
Water	309	50
B-factors		
Protein	24.532	66.179
Zinc	-	69.900
Sodium	-	53.805
Glycerol	33.653	-
Water	43.867	50.887
R.m.s. deviations		
Bond lengths (Å)	0.006	0.007
Bond angles (°)	1.147	0.940

Data were collected from a single crystal. Values in parentheses are for the highest resolution shell.

The co-crystallization of SEE and TCR was carried out by vapour diffusion at an equimolar ratio of the two proteins at 6.5 mg/ml total protein concentration, in 15% PEG 20,000, 0.1 M glycine pH 9.0 and 0.1 M NaCl. Crystals were soaked in cryo protectant containing 20% glycerol and flash-frozen in liquid nitrogen. Data of the SEE-TCR complex were collected at ESRF beamline ID 23–1 at a wavelength of 0.9763 Å and 100 K, with 0.5° oscillation, from a single crystal (Table 1). The data were processed using XDS [23] and aimless [24, 25] within the CCP4 suite [37] with 5% of the data chosen as a subset for cross-validation. The SEE-TCR structure was solved using the TCR structure presented here and a polyalanine model of SEE, generated from the SEA structure 1ESF [16] in Phaser [27], and amino acids were built manually in Coot [34]. Refinement was carried out using refmac5 [33] and autoBUSTER [32], with automatically generated TLS groups, and manual modeling was done in Coot [34]. A composite omit map was generated using CNS [35] with 5% of the structure omitted. The model comprised residues 3–190 in TCRα, 3–241 in TCRβ, and 10–233 in SEE, apart from residues 94–97 in TCRα, 183 in TCRβ, and 44–49 in SEE. In addition, the model includes 50 water molecules, two sodium ions and one zinc ion. Ramachandran statistics, calculated with SFCHECK [36], were 89% in preferred, 10.3% in allowed, and 0.7% in generously allowed regions.

### Computational modeling using Rosetta Dock

The initial TCR-SEE-(MHC)_2_ model was generated by modelling the missing residues 44–49 in SEE according to the SEA^D227A^-MHC structure (1LO5) [15]. The MHC structure and its position with respect to the SAg in the SEA^D227A^-MHC structure was used to generate the TCR-SEE-MHC model and side chains were energy minimized with the Rosetta Docking Prepack Protocol. Constraints based on the SEA^D227A^-MHC interface (S1 Table) were placed and 5000 models were generated using the high resolution protocol in Rosetta Dock [38–41]. Models were scored based on the interface between SEE-TCR and MHC and their RMSD from the starting model (S1 Fig), and the model with lowest interface score was chosen and refined by generating 1000 additional models, again with the high resolution protocol only. This was repeated, and the final model chosen was tested by running an unconstrained docking simulation according to the full Rosetta Dock protocol, which revealed a funnel-like shape of the plot of interface score versus RMSD for the models (S1 Fig), suggesting that the final model is energetically favourable, given that the MHC is located approximately in that region. For the high affinity zinc-bridged MHC site, the MHC molecule from the SEH-MHC class II structure (1HXY) [14] was placed according to the position of the MHC with respect to the SAg in the SEI-MHC structure (PDB: 2G9H) [42], this due to the identical conservation of the zinc site between SEE and SEI. The zinc-coordinating residues, His187, His225 and Asp227 in SEE, as well as His81 in MHCβ were constrained with respect to the Zn^2+^ and due to the conservation of Gln135 between SEE and SEH, this residue was constrained to contact the peptide as seen in the SEH-MHC structure [14] (S1 Table). Modelling of this site was carried out in the same manner as for the low-affinity site (S2 Fig). The models of the low and high affinity sites were then aligned and combined to a single TCR-SEE-(MHC)_2_ model.

## Results and Discussion

### Overall structure of the T cell receptor

The crystal structure of extracellular domains of a human chimeric TCR was determined to 1.35 Å resolution. The TCR was crystallized in space group P2_1_ with one molecule in the asymmetric unit. Data collection and refinement statistics are summarized in Table 1. The variable domains TRAV22 and TRBV7-9 were isolated from two TCRs specific against HLA-A2 in complex either with a telomerase peptide (sequence ILAKFLHWL) or a survivin peptide (sequence ELTLGEFLKL), respectively. The TCR exhibited the conventional TCR fold described previously [2]. Briefly, the TCR is a heterodimeric protein consisting of an α- and a β-chain, each with one membrane-distal variable domain (TRAV and TRBV) and one membrane-proximal constant domain (TRAC and TRBC). All four domains have Ig-like folds, almost exclusively consisting of β-sheets. The variable domains have four loops each, CDR1-3 and HV4, and together this surface is responsible for pMHC recognition.

### The structure of TCR in complex with staphylococcal enterotoxin E

**Fig 1:**
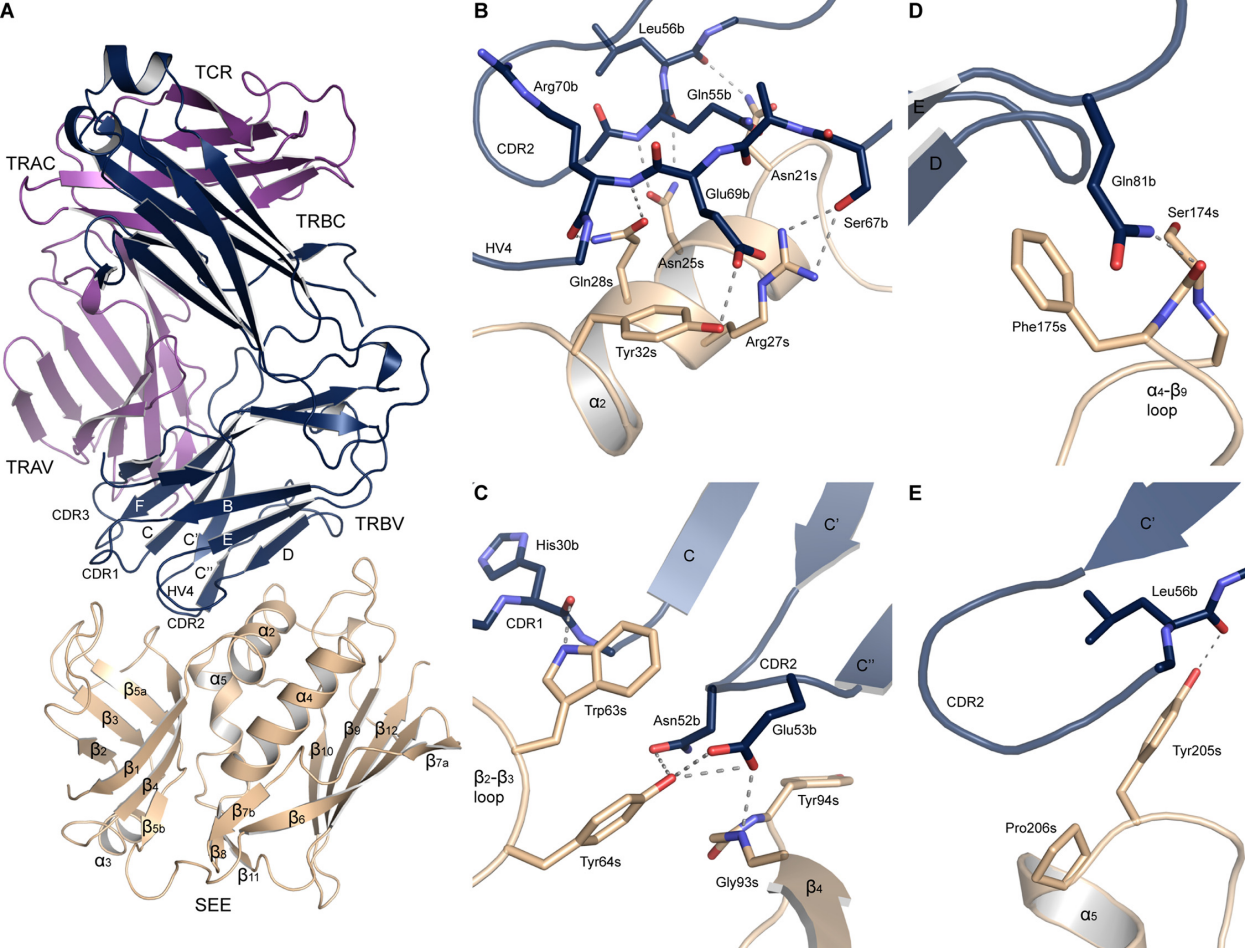
X-ray structure of the SEE-TCR complex. (A) Overall structure of the complex, with SEE in beige, the TCR α-chain in purple and the β-chain in blue. (B) Close-up of the SEE α_2_-helix and contacting residues in TCR, (C) the hydrophobic patch, (D) the α_4_-β_9_ loop, and (E) the upper part of the α_5_-helix. Hydrogen bonds are marked as dotted lines.

In order to study superantigen recognition by TCR, the structure of the TRAV22/TRBV7-9 TCR was determined in complex with staphylococcal enterotoxin E, to a resolution of 2.5 Å (Fig 1A). The complex crystallized in space group P2_1_2_1_2_1_ with one protein complex in the asymmetric unit. Data collection and refinement statistics are summarized in Table 1. The T cell receptor exhibited the same fold as described in the previous section, and the superantigen shares a similar fold with other bacterial superantigens, as first described for SEB [43]. SEE consists of two domains, an N-terminal domain resembling an oligosaccharide binding fold and a C-terminal β-grasp motif. The N-terminal domain consists of β-sheets (β_1_-β_5_) and a short α-helix (α_3_), and the C-terminal domain consists of an antiparallel β-sheet (β_6_, β_7_, β_9_, β_10_, and β_12_) packed against three α-helices (α_2_, α_4_ and α_5_), as well as a small two-stranded β-sheet (β_8_, β_11_). SEE is structurally and sequentially similar to SEA, with RMSD values between main chain atoms of 0.79 and 0.77 for the respective copies of SEA in the published three-dimensional structure (PDB ID: 1ESF) [16], and a sequence identity of 82%. In general, SEE engages the TRBV domain of TCR with the TCR binding site described for most other bacterial superantigens (Fig 1) [7, 44]. In the following paragraphs, residues will be designated a for TCRα, b for TCRβ, and s for SEE.

### The interface between TCR and staphylococcal enterotoxin E

**Fig 2:**
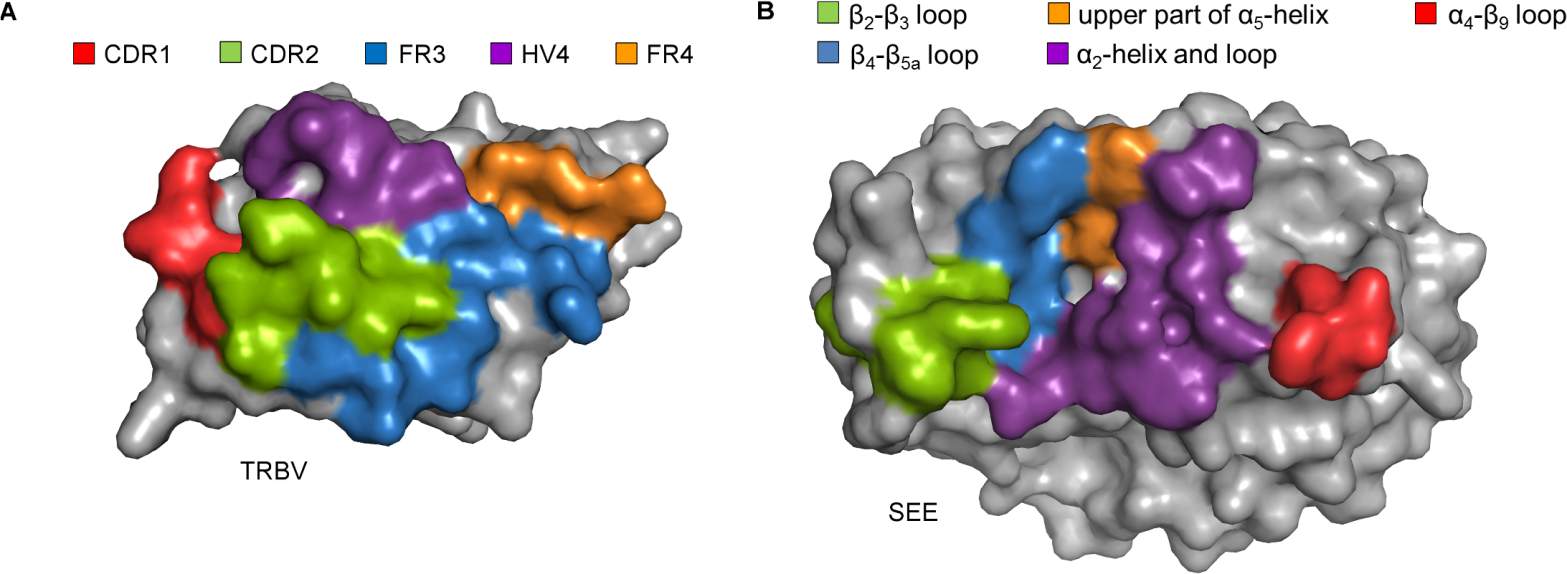
Presentation of the buried surface areas in the SEE-TCR interface. (A) The areas in the TRBV domain which are buried upon binding are marked in colors corresponding to the CDR1 loop (red), CDR2 loop (green), FR3 region (blue), HV4 loop (purple) and FR4 region (orange). (B) The buried surface area in SEE is marked in colors corresponding to the α_2_-helix and following loop (purple), the hydrophobic patch consisting of the β_2_-β_3_ (green) and β_4_-β_5a_ (blue) loops, the α_4_-β_9_ loop (red), and the upper part of the α_5_-helix (orange).

The T cell receptor recognizes SEE using its TRBV domain by binding to a shallow groove between the N- and C-terminal domains of SEE. The interface buries a dual surface area of 1945 Å^2^, with main contributions from the CDR2 loop (38.4%), the FR3 region (24.3%) and the HV4 loop (20.8%), as well as smaller contributions from the CDR1 loop (8.4%) and FR4 region (8.1%), whereas there are no contacts to the CDR3 loop of TRBV (Fig 2A). There are 16 hydrogen bonds present (Fig 1, S2 Table), and Van der Waals contacts (≤ 4.0 Å) are made with 23 residues in TCR and 19 in SEE (S3 Table). There are four major TCR-contacting points in the SAg: the α_2_-helix, the hydrophobic patch consisting of the β_2_-β_3_ and β_4_-β_5a_ loops, the α_4_-β_9_ loop, and the upper part of the α_5_-helix (Fig 1 and Fig 2B). Residue Asn25s, located in the α_2_-helix, which also is important for T cell activation in other superantigens [45–47], forms two hydrogen bonds to the backbone of Gln55b in the CDR2 loop (Fig 1B). In addition, residues Asn21s, Arg27s, and Gln28s, also in the α_2_-helix, form hydrogen bonds to CDR2 and HV4 (Fig 1B). Interestingly, five out of eight hydrogen bonds from the α_2_-helix are to the backbone of the TCR (Fig 1B, S2 Table). The hydrophobic patch consists of Gly93s and Tyr94s in the β_4_-β_5a_ loop, and is extended with residues Trp63s and Tyr64s in the β_2_-β_3_ loop. In particular, Trp63s packs against the CDR1, 2 and HV4 loops and contributes alone to 14% of the buried surface area of SEE (Fig 1C). In contrast to the α_2_-helix of SEE, the hydrophobic patch forms hydrogen bonds mostly to side chain atoms of TRBV, for instance by Tyr64s and Gly93s to the CDR2 loop (Fig 1C). Another feature of the SEE-TCR interface is the α_4_-β_9_ loop, which packs against the upper parts of the FR3 and FR4 regions. Here, Phe175s contributes with 13% to the buried surface area in SEE, and Ser174s forms a hydrogen bond to the side chain of Gln81b (Fig 1D). Lastly, the N-terminal part of helix α_5_ is also involved in the interface, utilizing residues Tyr205s and Pro206s, where a hydrogen bond is formed by Tyr205s to the main chain of Leu56b in FR3 (Fig 1E).

There are six previously determined superantigen structures from S. aureus with TCR available in the Protein Data Bank (SEB, SEC3, SEG, SEH, SEK and TSST-1), and two from *S*. *pyogenes* (SPE-A and SPE-C) [6, 44, 48–52]. A structural alignment using PROMALS3D was preformed to investigate whether the residues that are important for TRBV recognition in SEE are conserved among the other superantigens [53]. As shown in S3 Fig, most of the residues in the SEE-TCR interface are not conserved among the other SAgs (S3 Fig). However, in seven out of the nine structures investigated, the corresponding residue to Asn25s is present and forms hydrogen bonds to TCR in their respective structures. Moreover, Tyr64s is rather conserved (five out of nine) but is not involved in binding TCR in the other structures. Taken together, SEE utilizes, to a large extent, different amino acids to bind TCR compared to the previously determined SAgs, except for Asn25s. This correlates well with little overlap in TRBV specificity between SEE and the other SAgs [54]. However, superantigens in group III, as SEA, will possibly have a more SEE-like TCR recognition interface.

**Fig 3:**
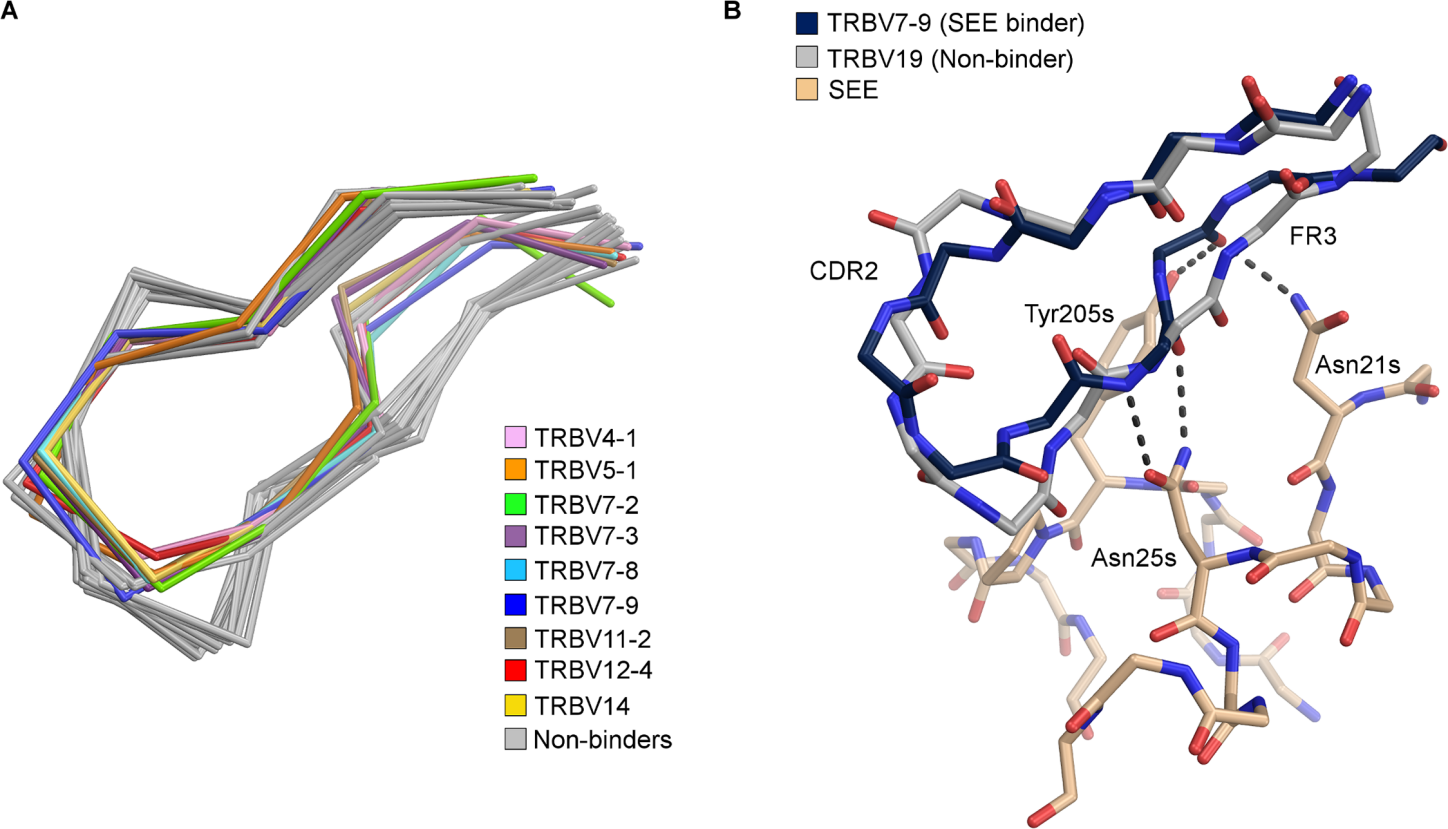
Comparison between structurally determined CDR2 loops. (A) Close-up view of the CDR2β loop of 23 structurally determined TCRs. TRBV domains with coloured loops have been reported to bind SEE, while the non-binders are shown in grey; TRBV4-1 (pink), TRBV5-1 (orange), TRBV7-2 (green), TRBV7-3 (purple), TRBV7-8 (cyan), TRBV7-9 (blue), TRBV11-2 (brown), TRBV12-4 (red), and TRBV14 (yellow), (B) comparison between the SEE-TRBV7-9 structure in beige and blue, respectively, and TRBV19 in grey, which do not bind SEE. The hydrogen bond pattern to the backbone of CDR2 and the C” strand is shown with dotted lines.

Staphylococcal enterotoxin E has a rather broad TRBV profile, and activates T cells bearing TRBV4, 5-1, 7, 11, 12, 14, 15, and 18 [11]. Commonly, the CDR2 loop and C” strand can adopt two different conformations in TRBV domains. The CDR2 loops and C” strands from 23 different structurally determined TRBVs [28, 29, 55–73], with either of these two conformations, were aligned (Fig 3A). Notably, eleven of these TRBV domains share the same C” strand conformation as TRBV7-9, whereas the TRBV domains that are not activated by SEE adopt the other conformation (Fig 3A). Clearly, the hydrogen bond pattern formed by SEE to TRBV7-9 cannot be formed to TRBV domains exhibiting the other conformation, as exemplified by TRBV19 (Fig 3B). Eight out of the eleven with the same CDR2 loop and C” strand conformation, are activated by SEE. Due to the rather broad reactivity of SEE and the common CDR2 structure of TRBVs recognized by SEE, it is likely that this SAg discriminates between TRBV domains partly based on CDR2 loop conformation (Fig 3A) [11]. However, three TRBV domains with the same CDR2 conformation are still not recognized by SEE: TRBV9, TRBV5-6 and TRBV5-8 (Fig 3A) [11]. Another important player for the TRBV specificity for SEE is the HV4 loop. It has been shown that T cells expressing the TRBV7-2*01 allele, with a Gly84b (IMGT numbering) in the HV4 loop [63], which corresponds to Gly73b in TRBV7-9, are activated by SEE, while TRBV7-2*02 with a glutamate instead of a glycine, is not activated by SEE [5]. Hence, it is likely that a large, charged amino acid in this position negatively affects the binding of SEE to TRBV. TRBV9 has an aspartate in this position [66], and thus this amino acid likely contributes to the ruling out of SEE recognition. Furthermore, TRBV9, TRBV5-6, and TRBV5-8 all have a glutamate at position 64 (IMGT numbering), in the CDR2 loop [57, 58, 66], whereas the corresponding residue in TRBV7-9 is Ala54b. This side-chain points towards residues Tyr205s and Pro206s in SEE. Thus, substituting the alanine to a glutamate would result in a negatively charged residue in a hydrophobic environment. In addition, the neighboring residue Asp207s may provide a negative charge, resulting in repulsion of a glutamate. Also, this region including both Pro206s and Asp207s, have been confirmed to be crucial for determining the TRBV specificity of SEE [74].

### Structural rearrangement in the TCR upon enterotoxin binding

**Fig 4:**
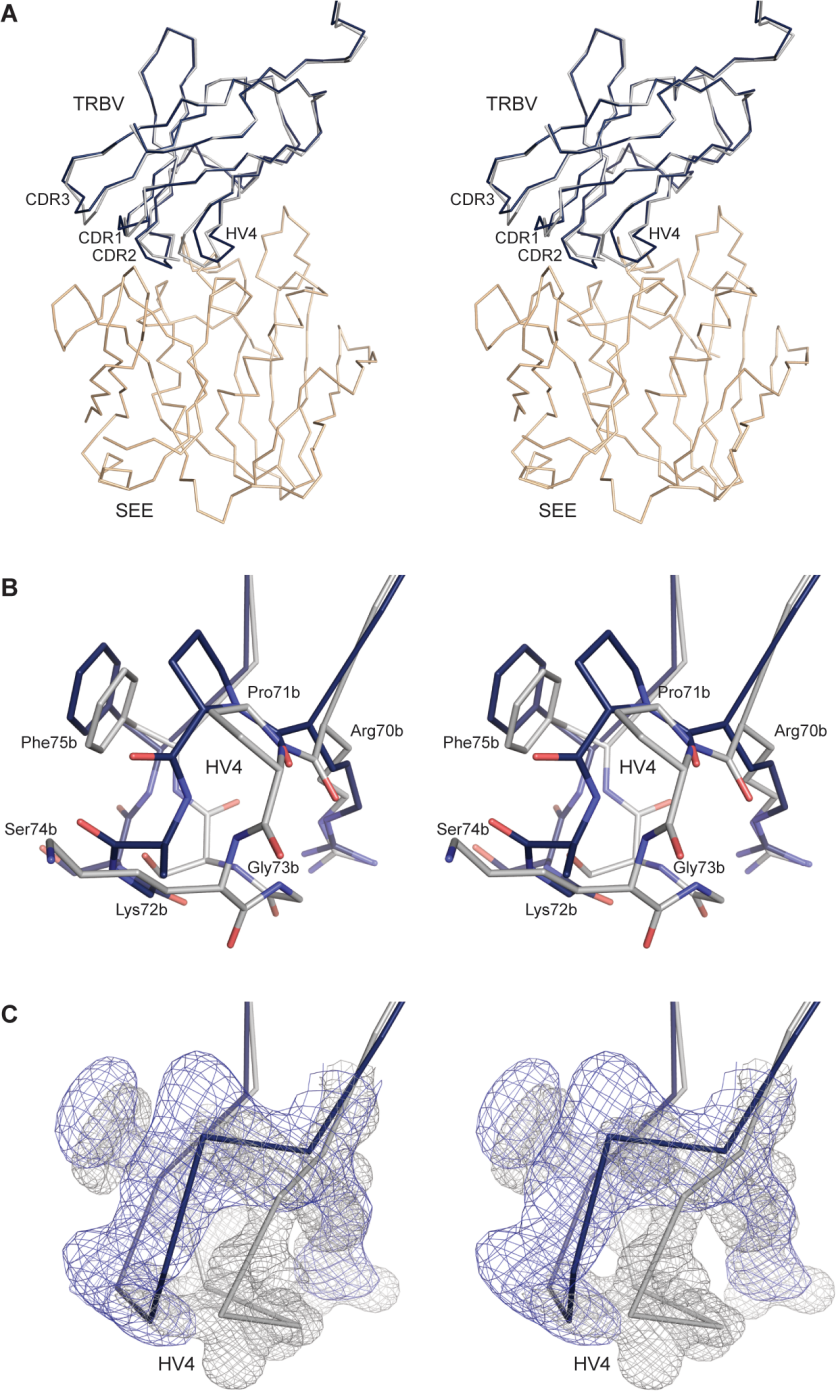
Comparison between the TCR and SEE-TCR structures, aligned with respect to the TRBV domain, in cross-eyed stereo view. (A) Overall differences between the TRBV domains, with the SEE-TCR structure shown in beige and blue and the TCR structure in grey. (B) Close-up of the HV4 loop with residues shown as sticks. (C) Close-up of the HV4 loop shown as Cα trace with 2F_o_-F_c_ electron density maps shown for the two structures, with the SEE-TCR map in blue and the TCR map in grey.

Differences in the overall structure of the unbound and SEE-bound TCR are small, with RMSD for main chain atoms of 1.3 and 0.91 for TCRα and TCRβ, respectively. This might be due to the introduced disulfide bond between the TRAC and TRBC domains, which potentially could lock the constant domains in certain positions and thus inhibit potential conformational changes upon binding [75]. In the TRAV domain, there are no large changes in the loop conformations. The majority of the loop rearrangements occur in the TRBV domain, with the HV4 loop and CDR1 loops in different positions, whereas CDR2 is only slightly shifted (Fig 4A).

The largest change upon SEE binding is seen in the HV4 loop, which previously has been suggested to be of importance for SAg engagement [4, 5]. This loop undergoes a conformational change (Fig 4B and 4C), as indicated by with an RMSD of 2.4 Å for residues 69–74 between bound and unbound TCR, aligned with respect to the Cα atoms of the TRBV domains. The loop moves away from the superantigen upon complex formation to avoid steric clashes (Fig 4). The largest movement in the HV4 loop occurs at Gly73b with a magnitude of 4.3 Å. Subsequently, this results in two hydrogen bonds both between Arg70b and Gln28s, as well as van der Waals contacts from Arg70b to Tyr32s and Trp63s (S2 and S3 Tables). As mentioned above, the corresponding glycine in TRBV7-2*01 (Gly84, IMGT numbering) [63] has previously been shown to be involved in TRBV specificity for SEE [5]. When substituting TRBV7-2*01 to TRBV7-2*02, with a Gly84Glu substitution, SEE could no longer activate the T cells. This is likely due to a combination of electrostatic repulsions caused by Glu34s, which is located close to the HV4 loop and steric hindrance because of a restrained HV4 loop upon substitution [5]. Moreover, Glu69b adopts a single conformation in the bound TCR structure instead of a dual, as in the unligated TCR, since one of the conformations clashes with Tyr32s in SEE. The single conformation allows Glu69b to interact with Arg27s, Gln28s and Tyr32s (S2 and S3 Tables). Pro71b is significantly shifted in order to accommodate for SEE, resulting in van der Waals contacts with Tyr32s. In contrast to this, no conformational changes have previously been seen in the HV4β loop upon SAg engagement in other studied TCR-SAg complexes (SEB, SEC3, SPE-A and SEG) [44, 48, 50, 52, 56, 76]. There are two sets of structures available with TCRs bearing the same TCR as studied here (TRBV7-9), alone and in complex with pMHC. An analysis of differences within each pair, aligned with respect to the Cα atoms of the TRBV domains, reveals no movements in the HV4 loop [29] [55]. Taken together, this suggests that the flexibility and hence the possibility to move the HV4 loop is of importance for SEE recognition, but is necessarily not directly coupled to general T cell activation by superantigens nor by conventional antigens.

### Model of the quaternary TCR-SEE-(MHC)_2_ complex

**Fig 5:**
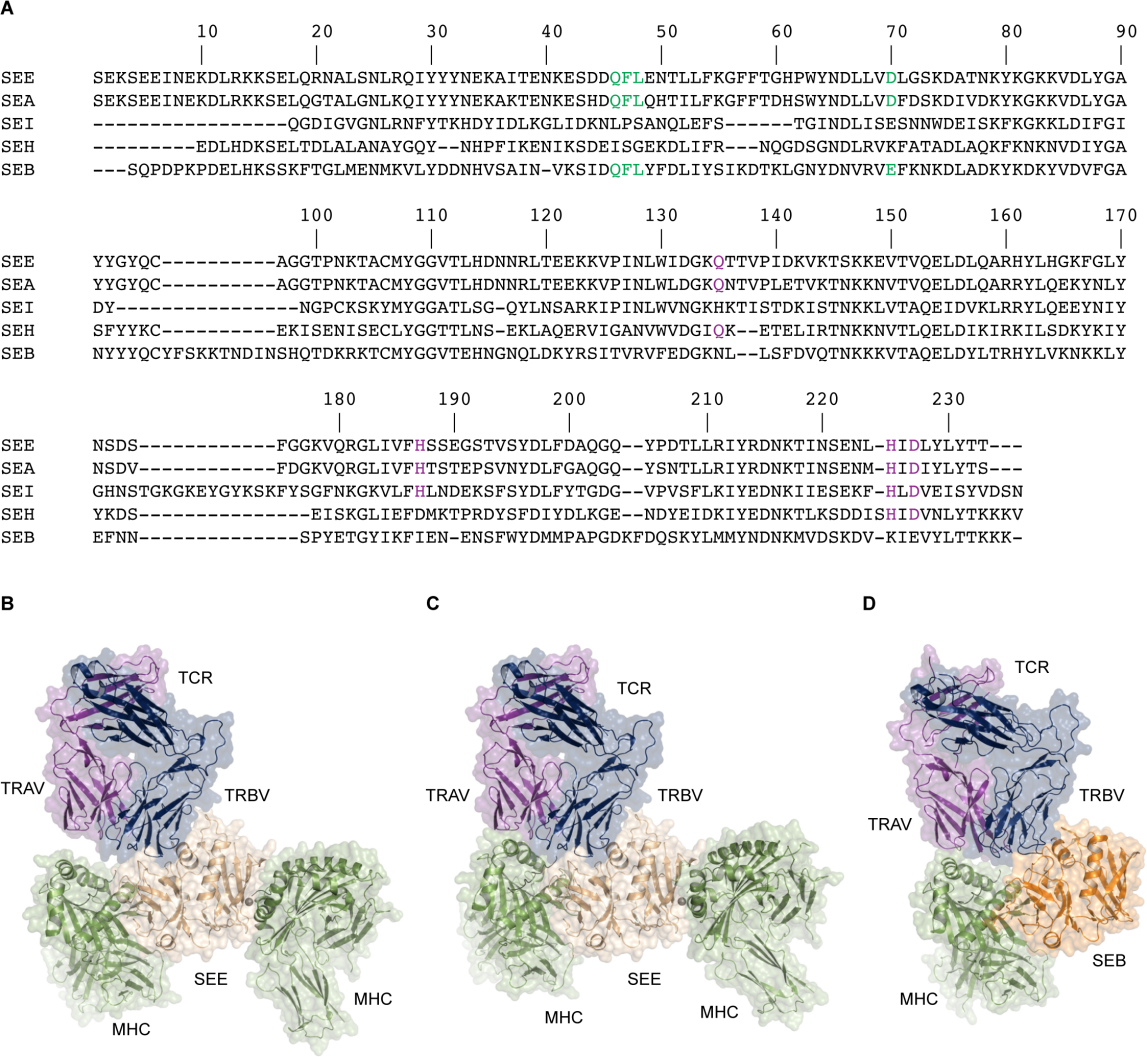
Modelling of the TCR-SEE-(MHC)_2_ quaternary complex. (A) Sequence alignment of SEE with SEA, SEB, SEH and SEI, displaying the conservation of both MHC binding sites in SEE, made using ClustalW2 [84, 85]. The N-terminal binding site to the MHC α-chain is marked in green and the C-terminal binding site to the MHC β-chain is marked in purple. (B) The initial model of TCR-SEE-(MHC)_2_. The TCR is shown in purple and blue (TCRα and TCRβ respectively), the SEE in beige, and MHC molecules in green. (C) The final model of TCR-SEE-(MHC)_2_. (D) The TCR-SEB-MHC structure, with SEB shown in orange.

As mentioned, the group III SAgs is distinguished by one TCR binding site and two MHC class II binding sites [12–15]. One site is between the N-terminal domain of the SAg and the α-chain of MHC utilizing Lys39 on MHC class II [15], and the other is in the C-terminal domain of the SAg to the β-chain of MHC, bridged by a zinc ion utilizing His81 on MHC class II [14]. In contrast, the group II SAgs, such as SEB, only has one TCR and one MHC class II binding site, to the α-chain of MHC [37]. Due to the similarity between SEE and SEA in their dependence of His81 and Lys39 for MHC binding, and the sequential conservation of both the N-terminal and zinc dependent C-terminal MHC class II binding sites (Fig 5A), it is likely that SEE, as SEA, is able to cross-link two MHC class II molecules, as also previously been suggested (Fig 5B) [15, 77, 78]. A zinc ion is visible in the SEE-TCR structure presented here, coordinated by residues His187s, His225s, and Asp227s, as well as Asp225b from the β-chain of a symmetry-related TCR, due to crystal packing. The three equivalent residues in SEA, SEI and two in SEH are known to coordinate the zinc ion in these SAgs [42, 79, 80] and are crucial for biological activity [14, 81]. Since the zinc binding site to MHC is conserved (Fig 5A), it is likely that SEE will engage the β-chain of MHC, using its C-terminal domain, in a manner similar to what has been observed for SEI [42]. In addition, many of the known MHC-coordinating residues in the N-terminal MHC binding site (to the α-chain) are conserved between SEE, SEA and SEB (Fig 5A).

Recently, the three-dimensional structure describing the ternary complex of TCR-SEB-MHC was published [19]. The structure clearly showed that in addition to contacts between the superantigen and the TRBV domain, the TRAV domain of TCR contributed to the complex formation by contacting the MHC class II molecule directly. This supports previous findings by Andersen and co-workers, who proposed that there is an interface between the TRAV domain of TCR and MHC, upon SEC3 binding [82]. Since SEE has a similar MHC binding site as SEB, a putative contribution from the TRAV domain upon complex formation is not unlikely. This is supported by previously published data where the particular sequence of the TRAV domain influenced the level of T cell activation by SEE in cells expressing in MHCβH81Y [83]. By combining the available structure of the superantigens SEA^D227A^-MHC (PDB ID: 1LO5) [15] and the SEI-MHC (PDB ID: 2G9H) [42] as well as the SEH-MHC structures (PDB ID: 1HXY) [14], we were able to build an initial structural model of a TCR-SEE-(MHC)_2_ complex. This was then used to generate models of the complex using Rosetta Dock (Fig 5B, S1 Fig, S2 Fig and S1 Table) [38–41]. The initial model showed distances larger than 7.5 Å between the TRAV domain and the MHC β-chain, suggesting that the TRAV may not be able to have a stabilizing effect on ternary complex formation (Fig 5B). However, after running docking simulations, the final model displayed a pMHC shifted towards the TRAV domain resulting in a contact area between these proteins (Fig 5C). This is in line with what has been observed for SEB, where these domains can interact (Fig 5D) [19]. Thus, it is plausible that this TRAV-MHC contact is able to form in solution for SEE as well. Residues that are involved in the TRAV-MHCβ model interface are Asp66, Glu69, Gln70, Arg72, Ala73, Asp76 and Thr77 in the MHC β-chain, to mainly the CDR2 loop of the TRAV domain, but contacts to HV4 and CDR1 are also present. Notably, all of these residues except Gln70 and Arg72 are in contact with TRAV in the TCR-SEB-MHC structure [19]. Compared to the SEA^D227A^-MHC structure, our modeled SEE-MHCα interface buries the same surface area (approximately 1120 Å^2^) but has considerably fewer hydrogen bonds and the MHC has moved significantly. The TRAV-MHCβ interface buries approximately 1020 Å^2^ in total, which is more than double the size observed in the TCR-SEB-MHC structure. This is likely the cause for the quite large differences seen in the SEE-MHCα interface compared to the SEA^D227A^-MHC and SEB-MHC interfaces. It is also worth considering that no flexibility between SEE and TCR is allowed in the Rosetta model. In solution, flexibility in this region could allow for a more SEB-like position of MHC but still allowing for the larger TRAV-MHCβ interface. In line with these results, SEE has previously been suggested to activate T cells partly dependent on the TRAV domain, in addition to the clear specificity for the TRBV domain [83]. From our structural and modeling data, we can conclude that it is likely that an interface can be formed between the TRAV domain and MHCβ, and hence that the main reason for the observed TRAV specificity for SEE is a direct contact between the TRAV and the MHCβ, instead of the speculated indirect conformational changes of the TRBV domain [83].

## Conclusions

The structure presented here features staphylococcal enterotoxin E in complex with a T cell receptor. This structure, in combination with the unligated TCR structure, shows that flexibility of the HV4 loop is of importance for SEE binding to TCR. In addition, the structure suggests that SEE discriminates between TRBV domains primarily by a mechanism dependent on CDR2 loop conformation and HV4 loop flexibility, and secondarily by CDR2 and HV4 loop sequence. Lastly, a computer model of the TCR-SEE-(MHC)_2_ complex concludes that an interface between the TRAV domain of TCR and the MHC molecule, upon binding to the low affinity site of SEE, is possible and that it could stabilize ternary complex formation.

## Accession Numbers

Coordinates and structure factors have been deposited in the Protein Data Bank with accession numbers 4UDT and 4UDU for TCR and SEE-TCR, respectively.

## Supporting Information

S1 Fig MHC modeled at the N-terminal site of SEE-TCR. (A) Initial generation of 5000 models, plotted after interface score versus RMSD from the starting model. (B) Generation of 1000 models with the final model as starting point, to verify presence of a local energy minimum.
(PDF)

S2 Fig MHC modeled at the C-terminal, zinc bridged, site of SEE-TCR. (A) Initial generation of 5000 models, plotted after interface score versus RMSD from the starting model. (B) Generation of 1000 models with the final model as starting point, to verify presence of a local energy minimum.
(PDF)

S3 Fig Structure-based sequence alignment of superantigens. Superantigens that have been structurally determined with TCR were structurally aligned using PROMALS3D [53]. Amino acids important for TCR recognition for SEE are underlined, and conserved amino acids are marked in red.
(PDF)

S1 Table Constraints used for the Rosetta modeling of the MHC sites on the SEE-TCR structure. All constraints were set as atom pair constraints using a Gaussian functions with mean and standard deviations as specified. Residues are denoted s for SEE, α for MHCα, β for MHCβ and p for peptide.
(PDF)

S2 Table Hydrogen bonds in the SEE-TCR complex. (PDF)

S3 Table Intermolecular Van der Waals contacts (distances less than 4 Å) in the SEE-TCR complex. (PDF)
