# Supplementary material for: Structure of Staphylococcal Enterotoxin E in Complex with TCR Defines the Role of TCR Loop Positioning in Superantigen Recognition
Source: PLoS One. 2015 Jul 6;10(7):e0131988. doi: 10.1371/journal.pone.0131988 (PMC4492778; doi:10.1371/journal.pone.0131988)
Supplement: S1 Fig — (A) Initial generation of 5000 models, plotted after interface score versus RMSD from the starting model. (B) Generation of 1000 models with the final model as starting point, to verify presence of a local energy minimum. (PDF) [file pone.0131988.s001.pdf]

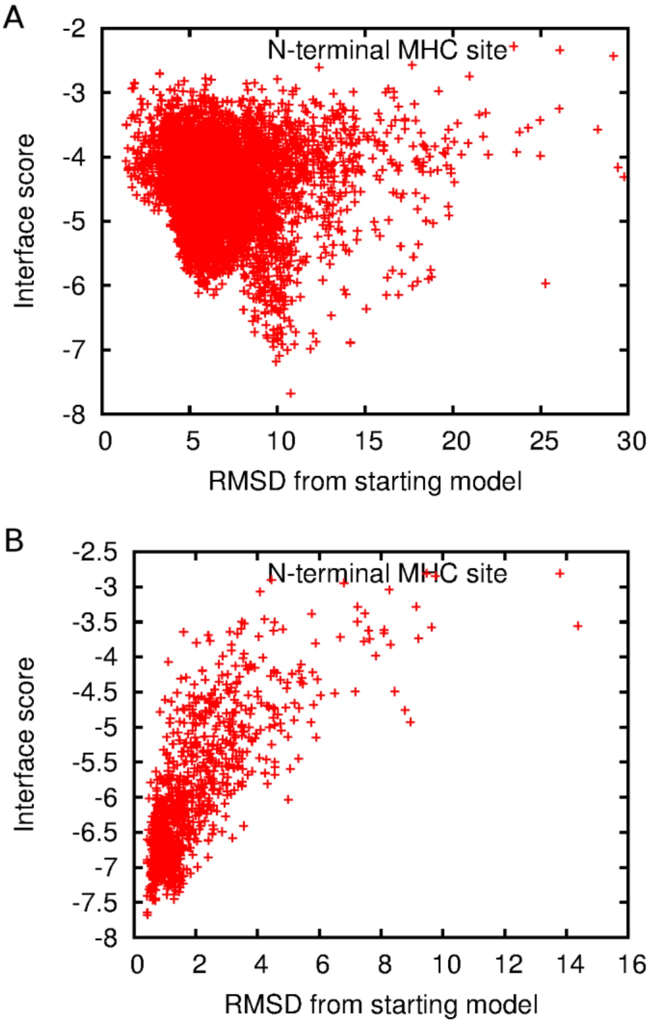


**S1 Fig. MHC modeled at the N-terminal site of SEE-TCR.** (A) Initial generation of 5000 models, plotted after interface score versus RMSD from the starting model. (B) Generation of 1000 models with the final model as starting point, to verify presence of a local energy minimum.
