# Supplementary material for: Structure of Staphylococcal Enterotoxin E in Complex with TCR Defines the Role of TCR Loop Positioning in Superantigen Recognition
Source: PLoS One. 2015 Jul 6;10(7):e0131988. doi: 10.1371/journal.pone.0131988 (PMC4492778; doi:10.1371/journal.pone.0131988)
Supplement: S3 Fig — Superantigens that have been structurally determined with TCR were structurally aligned using PROMALS3D [53]. Amino acids important for TCR recognition for SEE are underlined, and conserved amino acids are marked in red. (PDF) [file pone.0131988.s003.pdf]

```
                                  10          20        30        40        50        60
                                   |           |         |         |         |         |
SEE   (PDB ID:4UDU)       SEKSEEINEKDLRKKSELQ--RNALSNLRQIYYYNEKAITENKESDDQFLENTLLFKGFFTGHPWYNDLL
SEB   (PDB ID:1SBB)       -ESQPDPKPDELHKSSKFT---GLMENMKVLYDD-NHVSAINVKSIDQFLYFDLIYSIKDTKLGNYDNVR
SEC3  (PDB ID:1JCK)       -ESQPDPMPDDLHKSSEFT---GTMGNMKYLYDD-HYVSATKVKSVDKFLAHDLIYNINDKKLNNYDKVK
SEG   (PDB ID:3MC0)       ---QPDPKLDELNKVSDYKSNKGTMGNVMNLYMS-PPVEGRGVINSRQFLSHDLIFPIEY---KSYNEVK
SEH   (PDB ID:2XNA)       ---------EDLHDKSELT--DLALANAYGQYNH--PFIKENIKSDEISGEKDLIFRNQG---DSGNDLR
SEK   (PDB ID:2NTS)       -----------------QG--DIGIDNLRNFYTKKDFVDLKDVKDNDTPIANQLQFSN------ESYDLI
SPE-A (PDB ID:1L0X)       ---QQDPDPSQLHRSSLVK----NLQNIYFLYEG-DPVTHENVKSVDQLLSHDLIYNVSG---PNYDKLK
SPE-C (PDB ID:1KTK)       -------------DSKKDI--SNVKSDLLYAYTI-TPYDYKDCRV-NFSTTHTLNIDTQKYR-GKDYYIS
TSST-1(PDB ID:3MFG)       ---------------STN----DNIKDLLDWYSS-GSDTFTNSEVLD-NSLGSMRIKNTD----GSISLI

                          70        80        90        100                 110       120
                           |         |         |         |                   |         |
SEE                       VDLGSKDATNKYKGKKVDLYGAYYGYQCAGGT----------PNKTACMYGGVTLHDNNRLTEEKKVPIN
SEB                       VEFKNKDLADKYKDKYVDVFGANYYYQCYFSKKTNDINSHQTDKRKTCMYGGVTEHNGNQLDKYRSITVR
SEC3                      TELLNEDLANKYKDEVVDVYGSNYYVNCYFSSKDNV---GKVTSGKTCMYGGITKHEGNHFDNGNLQNVL
SEG                       TELENTELANNYKGKKVDIFGVPYFYTCIIPKSEPD---INQNFGGCCMYGGLTFNSSE-NERDKLITVQ
SEH                       VKFATADLAQKFKNKNVDIYGASFYYKCEKIS----------ENISECLYGGTTLNSE-KLAQERVIGAN
SEK                       SESKDFNKFSNFKGKKLDVFGISYNGQSN----------------TKYIYGGVTATNE-YLDKSRNIPIN
SPE-A                     TELKNQEMATLFKDKNVDIYGVEYYHLCYLSEN---------AERSACIYGGVTNHEGNHLEIPKKIVVK
SPE-C                     SEMSYEASQKFKRDDHVDVFGLFYILNSH---------------TGEYIYGGITPAQNNK--VNHKLLGN
TSST-1                    IFPSPYYSPAFTKGEKVDLNTKRTKKSQHTSE----------GTYIHFQISGVTNTEK--LPTPIELPLK

                          130       140       150       160       170                   180
                           |         |         |         |         |                     |
SEE                       LWIDGKQTTVPIDKVKTSKKEVTVQELDLQARHYLHGKFGLYNSDS------------FGGKVQRGLIVF
SEB                       VFEDGKN--LLSFDVQTNKKKVTAQELDYLTRHYLVKNKKLYEFN--------------NSPYETGYIKF
SEC3                      IRVYENKRNTISFEVQTDKKSVTAQELDIKARNFLINKKNLYEFN--------------SSPYETGYIKF
SEG                       VTIDNRQ--SLGFTITTNKNMVTIQELDYKARHWLTKEKKLYEFD--------------GSAFESGYIKF
SEH                       VWVDGIQK--ETELIRTNKKNVTLQELDIKIRKILSDKYKIYYKD---------------SEISKGLIEF
SEK                       IWINGNHKTISTNKVSTNKKFVTAQEIDVKLRKYLQEEYNIYGHNGTKKGEEYGHKSKFYSGFNIGKVTF
SPE-A                     VSIDGIQ--SLSFDIETNKKMVTAQELDYKVRKYLTDNKQLYTNG--------------PSKYETGYIKF
SPE-C                     LFISGESQQNLNNKIILEKDIVTFQEIDFKIRKYLMDNYKIYDAT---------------SPYVSGRIEI
TSST-1                    VKVHGKDSP-LKYWPKFDKKQLAISTLDFEIRHQLTQIHGLYRS----------------SDKTGGYWKI

                            190       200          210       220        230
                             |         |            |         |          |
SEE                       HSSEGSTVSYDLFDAQG---QYPDTLLRIYRDNKTINSENL-HIDLYLYTT---
SEB                       IENE-NSFWYDMMPAPGD-KFDQSKYLMMYNDNKMVDSKDV-KIEVYLTTKKK-
SEC3                      IESNGNTFWYDMMPAPGD-KFDQSKYLMIYKDNKMVDSKSV-KIEVHLTTKNG-
SEG                       TEKNNTSFWFDLFPKKELVPFVPYKFLNIYGDNKVVDSKSI-KMEVFLNT----
SEH                       DMKTPRDYSFDIYDLKG---ENDYEIDKIYEDNKTLKSDDISHIDVNLYTKKKV
SEK                       HLNNNDTFSYDLFYTGD--DGLPKSFLKIYEDNKTVESEKF-HLDVDISYKA--
SPE-A                     IPKNKESFWFDFFPEPE---FTQSKYLMIYKDNETLDSNTS-QIEVYLTTK---
SPE-C                     GTKDGKHEQIDLFDSPN--EGTRSDIFAKYKDNRIINMKNFSHFDIYLEK----
TSST-1                    TMNDGSTYQSDLSK-----------KFEYNTEKPPINIDEIKTIEAEIN-----
```

**S3 Fig. Structure-based sequence alignment of superantigens.** Superantigens that have been structurally determined with TCR were structurally aligned using PROMALS3D (Pei et al, 2008). Amino acids important for TCR recognition for SEE are underlined, and conserved amino acids are marked in red.


Pei J, Kim BH, Grishin NV (2008) PROMALS3D: a tool for multiple protein sequence and structure alignments. *Nucleic Acids Res* **36:** 2295-2300
