# Supplementary material for: Structure of Staphylococcal Enterotoxin E in Complex with TCR Defines the Role of TCR Loop Positioning in Superantigen Recognition
Source: PLoS One. 2015 Jul 6;10(7):e0131988. doi: 10.1371/journal.pone.0131988 (PMC4492778; doi:10.1371/journal.pone.0131988)
Supplement: S1 Table — All constraints were set as atom pair constraints using a Gaussian functions with mean and standard deviations as specified. Residues are denoted s for SEE, α for MHCα, β for MHCβ and p for peptide. (PDF) [file pone.0131988.s004.pdf]

S1 Table. Constraints used for the Rosetta modeling of the MHC sites on the SEE-TCR structure. All constraints were set as atom pair constraints using a Gaussian functions with mean and standard deviations as specified. Residues are denoted s for SEE, α for MHCα, β for MHCβ and p for peptide.

**Residue 1**	**Atom 1**	**Residue 2**	**Atom 2**	**Mean**	**S.D.**
Low affinity MHCα site					
Phe47s	Cε2	Leu60α	Cδ2	3.5	0.4
Phe47s	Cε1	Ala64α	Cβ	3.8	0.4
Phe47s	O	Lys67α	Nζ	3.2	0.4
Asp70s	Oδ1	Lys39α	Nζ	3.0	0.2
Asp70s	Oδ2	Lys39α	Nζ	3.5	0.4
High affinity MHCβ site					
Zn	Zn	His81β	Nδ1	2.1	0.1
Zn	Zn	His187s	Nδ1	2.1	0.1
Zn	Zn	His225s	Nε2	2.1	0.1
Zn	Zn	Asp227s	Oδ2	2.1	0.1
Gln135s	Oε1	Lys5p	N	3.0	0.4
Gln135s	Nε2	Lys5p	O	3.0	0.4
