# Supplementary material for: Structure of Staphylococcal Enterotoxin E in Complex with TCR Defines the Role of TCR Loop Positioning in Superantigen Recognition
Source: PLoS One. 2015 Jul 6;10(7):e0131988. doi: 10.1371/journal.pone.0131988 (PMC4492778; doi:10.1371/journal.pone.0131988)
Supplement: S2 Table — (PDF) [file pone.0131988.s005.pdf]

S2 Table. Hydrogen bonds in the SEE-TCR complex.

SEE		TRBV		
**Residue**	**Atom**	**Residue**	**Atom**	**Distance**
Asn21	Oδ1	Gln55	Oε1	3.0
Asn21	Nδ2	Leu56	O	2.8
Asn25	Oδ1	Gln55	N	2.8
Asn25	Nδ2	Gln55	O	3.1
Arg27	Nη1	Ser67	Oγ	3.1
Arg27	Nη2	Ser67	Oγ	2.8
Gln28	Oε1	Arg70	N	2.8
Gln28	Nε2	Arg70	O	3.1
Tyr32	Oη	Glu69	Oε1	2.7
Trp63	Nε1	His30	O	2.8
Tyr64	Oη	Asn52	Oδ1	3.1
Tyr64	Oη	Glu53	Oε1	3.1
Tyr64	Oη	Glu53	Oε2	2.7
Gly93	N	Glu53	Oε1	2.9
Ser174	O	Gln81	Nε2	2.8
Tyr205	Oη	Leu56	O	2.8
