# Supplementary material for: Structure of Staphylococcal Enterotoxin E in Complex with TCR Defines the Role of TCR Loop Positioning in Superantigen Recognition
Source: PLoS One. 2015 Jul 6;10(7):e0131988. doi: 10.1371/journal.pone.0131988 (PMC4492778; doi:10.1371/journal.pone.0131988)
Supplement: S3 Table — (PDF) [file pone.0131988.s006.pdf]

S3 Table. Intermolecular Van der Waals contacts (distances less than 4 Å) in the SEE-TCR complex.

SEE	TCR
Asn21	Ser28
Ser24	Glu29
Asn25	His30
Arg27	Asn31
Gln28	Gln51
Tyr32	Asn52
Asn33	Glu53
Pro62	Ala54
Trp63	Gln55
Tyr64	Leu56
Tyr91	Glu57
Tyr92	Lys58
Gly93	Asp64
Tyr94	Arg65
Ser172	Ser67
Ser174	Ala68
Phe175	Glu69
Tyr205	Arg70
Pro206	Pro71
	Lys72
	Glu79
	Gln81
	Arg82
